# Regularized Iterative Weighted Filtered Back-Projection for Few-View Data Photoacoustic Imaging

**DOI:** 10.1155/2016/9732142

**Published:** 2016-08-09

**Authors:** Xueyan Liu, Dong Peng

**Affiliations:** ^1^Department of Mathematics Science, Liaocheng University, Liaocheng 252000, China; ^2^Life Sciences Research Center, School of Life Sciences and Technology, Xidian University, Xi'an 710071, China

## Abstract

Photoacoustic imaging is an emerging noninvasive imaging technique with great potential for a wide range of biomedical imaging applications. However, with few-view data the filtered back-projection method will create streak artifacts. In this study, the regularized iterative weighted filtered back-projection method was applied to our photoacoustic imaging of the optical absorption in phantom from few-view data. This method is based on iterative application of a nonexact 2DFBP. By adding a regularization operation in the iterative loop, the streak artifacts have been reduced to a great extent and the convergence properties of the iterative scheme have been improved. Results of numerical simulations demonstrated that the proposed method was superior to the iterative FBP method in terms of both accuracy and robustness to noise. The quantitative image evaluation studies have shown that the proposed method outperforms conventional iterative methods.

## 1. Introduction

Photoacoustic imaging (PAI) combining good acoustic resolution with high optical contrast in a single modality has great potential for tremendous clinical applications [1]. It is promising in many aspects, for example, the detection of breast cancer, skin cancer, and osteoarthritis in humans [2–4]. In the past decades, many algorithms have been proposed for image reconstruction when the ultrasonic transducer collects signals from a full view [5–7]. A limiting factor for these algorithms is a large number of measurements made with transducers. In addition, in many potential applications of PAI, such as ophthalmic imaging and breast imaging, the object is only accessible from limited angles. A practical need exists for reconstruction from few-view data, as this can greatly reduce the required scanning time and the number of ultrasound sensors [8–11].

Analytic algorithms like filtered back-projection (FBP) and time-reversal based reconstruction attain very fast reconstruction performance [5, 12]. However, these algorithms have an inherent limitation of requiring large number of data points around the target object for accurately estimating the optical absorption. And implementations of such formulae may cause streak-type artifacts and negative values in the reconstructed image. To overcome these limitations, iterative image reconstruction algorithms have been proposed to improve the reconstructed image quality [13–16], which can mitigate artifacts from incomplete few-view data and permit reductions in data-acquisition times. And iterative methods can be significantly accelerated with GPU-based reconstructions [17]. Because of this, the development of iterative image reconstruction algorithms for PAI is an important research topic of current interest. By minimizing the least-square error between the measured signals and the signals predicted by the exact photoacoustic propagation model, the model-based photoacoustic inversion method has been proved to be stable and accurate. However, its reconstruction is computationally burdensome which limits its application in the practical PAI [16–18]. Finally, iterative weighted algorithms can effectively mitigate image artifacts due to limited-view acoustic data [19–21]. All of these methods provide the opportunity for accurate image reconstruction from few-view data.

In this study, inspired by the iterative weighted approaches in CT [20, 22, 23], we derived a regularized iterative weighted FBP (RIWFBP) method to improve the convergence properties of the iterative loop and improve image quality in few-view PAI. During the reconstruction, we firstly use the effective few-view scanning angle improved FBP method to reconstruct an initial image of the optical absorption [24]. In each iteration step, the difference between the collected signals and the calculated signals was used to update the correction image, and a regularization operation that improved the convergence properties of the iterative loop was added. Numerical simulation and experimental results reveal the good performance of the RIWFBP method.

This paper is organized as follows. In Section 2, the iterative improved FBP method and the regularized enhanced iterative scheme are reviewed briefly. In Section 3, besides using numerical phantoms, we also conducted experimental measurements and applied our reconstruction method to the obtained data. Finally, the conclusions are drawn in Section 4.

## 2. Methods

### 2.1. Iterative Improved Filtered Back-Projection Method

According to the forward problem for an acoustically homogeneous model present in [8], the acoustic wave pressure *p*(*r*_0_, *t*) at a detector position *r*_0_ and time *t* over a circle in the 2D imaging is related to the spatial distribution of electromagnetic absorption *A*(*r*),

(1) $$\begin{array}{c} p\left(\;r_{0},t\;\right)=\frac{c\beta I}{4\pi C_{p}}\frac{\partial }{\partial t}\overset{}{\underset{t=\left|r-r_{0}\right|/c}{\oint }}\frac{A\left(r\right)}{\left|r-r_{0}\right|}dr. \end{array}$$

Here *β* is the coefficient of volumetric thermal expansion; *C*_*p*_ is the isobaric specific heat; *c* is the speed of sound; and *I* is the temporal profile. For 2D imaging, the approximate inverse solution for the circular-scan geometry can be represented by [25]

(2) $$\begin{array}{c} A\left(\;r\;\right)=-\frac{r_{0}^{2}C_{p}}{2\pi \beta c^{4}}\int_{\varphi_{0}}d\varphi_{0}\frac{1}{t}{\left.\;\frac{\partial p\left(r_{0},t\right)}{\partial t}\;\right|}_{t=\left|r_{0}-r\right|/c}. \end{array}$$

To numerically model the above forward and inverse problems, we used vector *x* to represent *A*(*r*) and vector *y* to represent *p*(*r*_0_, *t*). Then the forward problem can be described as *y* = *Px*, and the reconstruction formula can be written as *x* = *RPx* [5], where *R* is the back-projection operator [8]. In real biological tissue imaging only the noisy signals can be detected from few-view data. *P* is an ill-conditioned matrix; thus we cannot obtain an exact image.

Iterative improved FBP (IFBP) methods have been used to reduce artifacts due to an insufficient data and streaks due to missing angles. The update step of IFBP is then given by [13]

(3) $$\begin{array}{c} x^{m}=x^{m-1}+\alpha R\left(\;y-Px^{m-1}\;\right),\;m=1,2,3,\ldots . \end{array}$$

In this way, a sequence of image vectors is produced.

### 2.2. Regularized Iterative Weighted Filtered Back-Projection Method

In this section, we will present the RIWFBP method for the few-view PAI imaging. This contribution is an extension of theory and experiments on iterative weighted FBP (IWFBP) presented in [26]. By using the effective scanning angle the algorithm for full-view data can be approximately extended to the few-view case. The reconstructed intensity error problem induced by few-view scanning can be improved [24]

(4) $$\begin{array}{c} A\left(\;r\;\right)\approx -\frac{r_{0}^{2}C_{p}}{2\pi \beta c^{4}}\overset{\theta_{2}}{\underset{\theta_{1}}{\int }}d\theta \frac{1}{\theta_{e}t}{\left.\;\frac{\partial p\left(r_{0},t\right)}{\partial t}\;\right|}_{t=\left|r_{0}-r\right|/c}, \end{array}$$

where *θ*_*e*_ is the effective scanning angle and *θ*_1_ and *θ*_2_ are, respectively, the minimum and maximum angle of the signal acquisition position. Based on (4), we used the RIWFBP method to compensate for the nonexactness of FBP.

During the reconstruction, we firstly used *θ*_*e*_ weighted FBP method to reconstruct an initial distribution *x* ^0^ of the absorbed energy density. Then we applied the RIWFBP method to update the distribution *x* ^*m*^ of the absorbed energy density. To compensate for the difference between the reconstruction *x* ^*m*^ and the actual image *x*, a weighted parameter [26],

(5) $$\begin{array}{c} \lambda_{m}=\frac{\max\left(y\right)-\min\left(y\right)}{\max\left(x^{m}\right)-\min\left(x^{m}\right)},\;m=0,1,2,\ldots , \end{array}$$

was used to correct the differences between the measured signals *y* and computed signals *Px* ^*m*^. We obtained the error correction image Δ*x* ^*m*^ from the differences between *y* and *λ*_*m*−1_ *Px* ^*m*^ at each iteration step. The recursion expression is as follows:

(6) xm=xm−1+Δxm=xm−1+αRy−λm−1Pxm−1,m=1,2,….

In practice, it might be wise to employ only a fraction *α* ∈ (0,1) of the full values of the correction image Δ*x* ^*m*^. Inspired by the idea of regularization [22, 23], the RIWFBP method is designed to reconstruct the absorbed energy deposition by adding a regularization operation in the iterative loop of IFBP. This is accomplished through the quadratic regularization for least-squares minimization of the following functional:

(7) $$\begin{array}{c} F\left(\;y,x\;\right)=\frac{1}{2}{\left\|\;y-Px\;\right\|}^{2}+\gamma \overset{N}{\underset{i=1}{\sum }}\overset{N}{\underset{j=1}{\sum }}d_{ij}{\left(\;x_{i}-x_{j}\;\right)}^{2}, \end{array}$$

where *γ* is a parameter determining the amount of regularization and *d*_*ij*_ are the inverse distances between the pixels *i* and *j* in a 3^2^ neighborhood. The last term is obviously a penalty term. The minimization of (7) can be realized by differentiating *F* with respect to *x*_*i*_ and setting each of the resulting expressions to zero, leading to the following system of equations:

(8) ∂F∂xi=PTy−Px+4γ∑i=1N∑j=1Ndijxi−xjei=0i=1,2,…,N,

where ∑_*i*=1_^*N*^(∑_*j*=1_^*N*^ *d*_*ij*_(*x*_*i*_ − *x*_*j*_))*e*_*i*_ = *Kx* [22] and {*e*_1_, *e*_2_,…, *e*_*N*_} is the standard basis for *R* ^*N*^. By using the steepest descent method the solution in an iterative form was given as

(9) $$\begin{array}{c} x^{m}=x^{m-1}-\alpha \left[\;P^{T}\left(\;y-Px^{m-1}\;\right)\;\right]-4\alpha \gamma Kx^{m-1}. \end{array}$$

Finally, the last term in (9) has been added to the update step in (6), resulting in

(10) $$\begin{array}{c} x^{m}=x^{m-1}+\alpha \left[\;R\left(\;y-\lambda_{m-1}Px^{m-1}\;\right)-\gamma Kx^{m-1}\;\right]. \end{array}$$

The convergence of the iteration has been explained by Sunnegårdh and Danielsson [22]. In this paper, the quality of the reconstructed image is measured via the normalized mean absolute error (NMAE), which is most sensitive to distortion artifacts defined as

(11) $$\begin{array}{c} \delta_{i}=\frac{{\left\|x_{i}-x\right\|}_{2}}{{\left\|x\right\|}_{2}}. \end{array}$$

After a few iterations, artifacts are suppressed while the edge and detailed information is preserved well. When a desired minimum NMAE has been achieved the iterative process will stop, and then the results will be output.

## 3. Results

### 3.1. Reconstructions from Simulated Few-View Data

**Figure 1:**
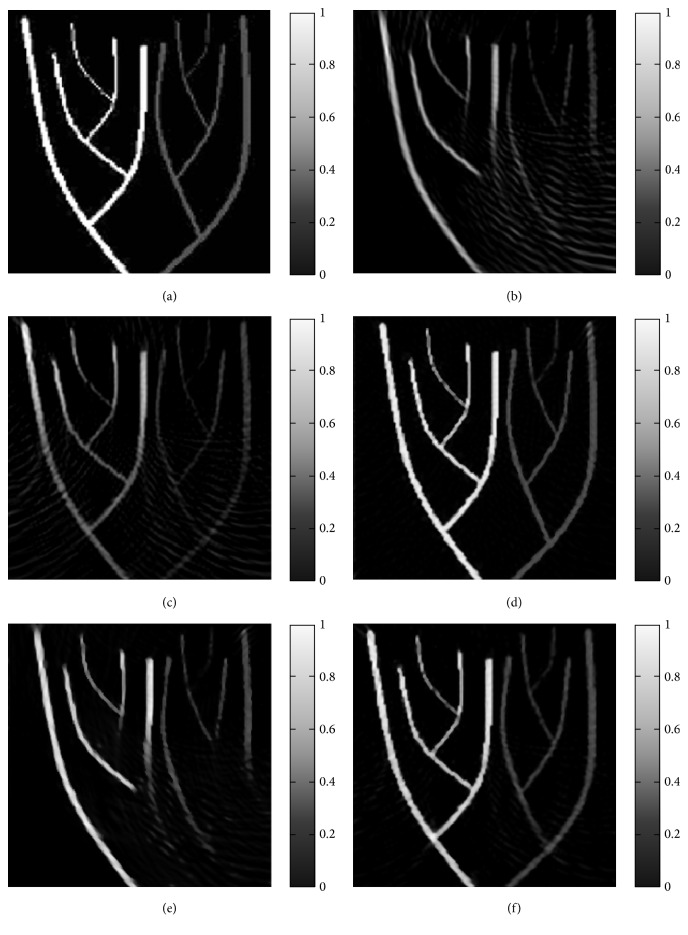
Images reconstructed from simulated data corresponding to the numerical phantom (a). (b) 30 detectors over 90°, using IFBP. (c) 60 detectors over 180°, using IFBP. (d) 120 detectors over 360°, using RIWFBP. (e) 30 detectors over 90°, using RIWFBP. (f) 60 detectors over 180°, using RIWFBP.

Computer simulations were conducted to demonstrate the effectiveness of the proposed method. The imaged source with a size of 256 × 256 pixels, as shown in Figure 1(a), was approximately located within a thin slab. The photoacoustic signals were calculated according to (1), and Gaussian noises were also added to simulated signals. In the experiments, all reconstruction methods were implemented in MATLAB (MathWorks, Natick, MA).

Images reconstructed with the IFBP method and the RIWFBP method from few-view data are displayed in Figure 1, respectively. There are many artifacts and blurs in Figures 1(b) and 1(c). We hypothesize that the distortions present in Figures 1(b) and 1(e) come from the limited information available. This result is in agreement with the theoretical prediction in [8]. When comparing Figures 1(d) and 1(f), it can be seen that the quality of the image reconstructed from few-view data is comparable with that from full-view. So we can conclude that with the incomplete data, the RIWFBP method can improve the quality of reconstruction with few-view data.

**Figure 2:**
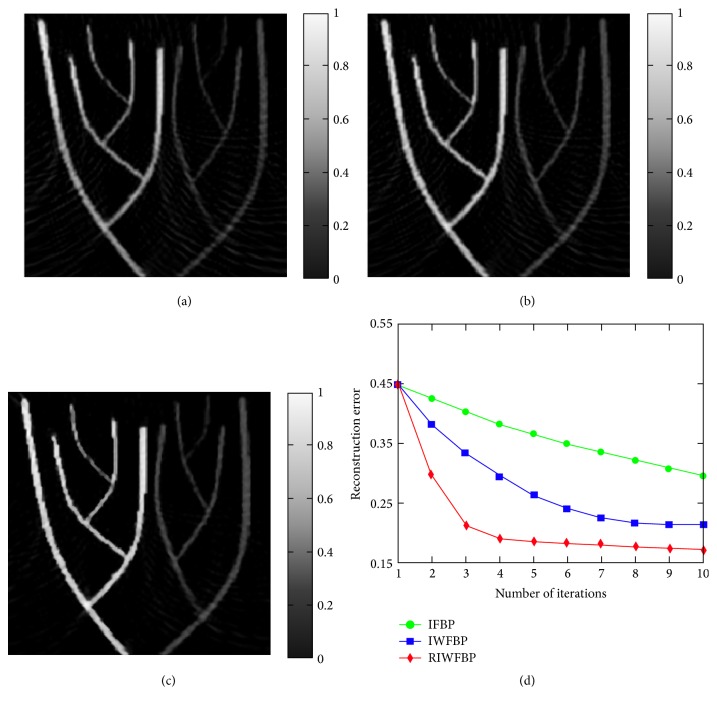
Photoacoustic imaging of the numerical phantom using 60 detections over 180°. (a) IFBP; (b) IWFBP; (c) RIWFBP. (d) Reconstruction normalized mean absolute error from 180° under different number of iterations.

Figures 2(a)–2(c) show the reconstructions utilizing the IFBP method, the IWFBP method [26], and the RIWFBP method with 60 detections over 180°, respectively. It is obvious that the artifacts clearly seen in Figure 2(a) are suppressed to a large degree in Figure 2(c). By choosing an appropriate value for the regularization parameter *γ*, the artifact characteristics of the FBP method can be preserved. The nonregularized IWFBP is clearly outperformed by the regularized.

Reconstruction errors between the original image and the reconstructed images were calculated and shown in Figure 2(d). We can see that the NMAE of the proposed method decrease especially when the iterative numbers are fewer. The reconstruction error for the RIWFBP falls to 0.18 in the first five iterations and continues to drop down to 0.17 after 10 iterations. In contrast to the IFBP method and the IWFBP method, the proposed method is expected to deliver a good result in less than five iterations.

**Figure 3:**
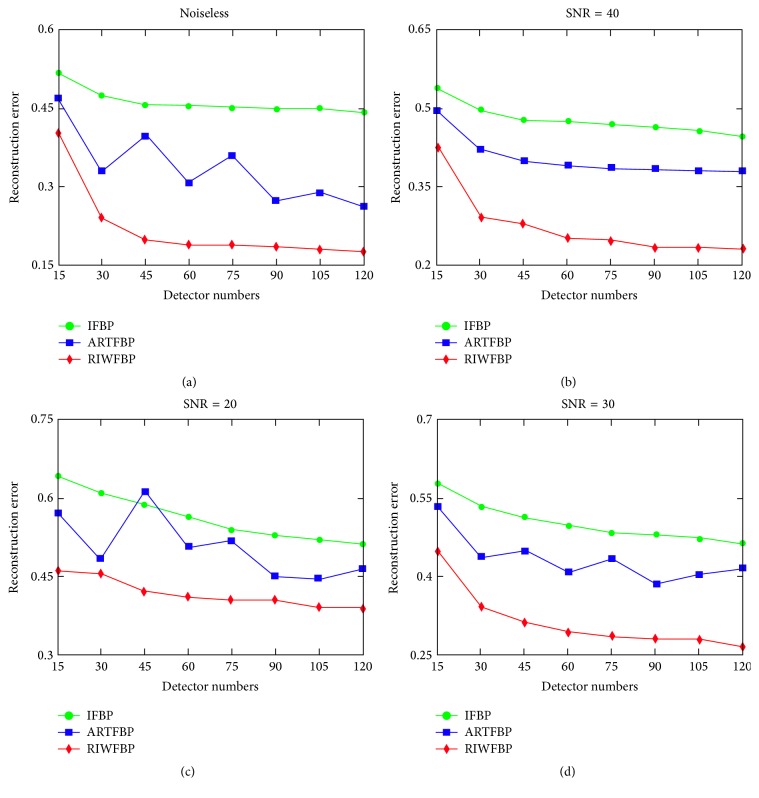
Reconstruction normalized mean absolute errors from 180° with a different detector number. (a) Noiseless observation; noisy observation with (b) SNR = 40 dB; (c) SNR = 30 dB; (d) SNR = 20 dB.

To study the robustness of the RIWFBP method, Gaussian noises with SNR 40, 30, and 20 were added to the signals.  Figure 3 shows the tendency chart of reconstruction errors between the phantom and the reconstruction obtained from the IFBP method, the ARTFBP method [27, 28], and the RIWFBP method. It can be demonstrate that the RIWFBP method has the minimum NMAE. The regularized iterative scheme is robust to inaccurate measurements and has greater improvement in calculation accuracy as predicted by the theory presented in [8]. The RIWFBP method can not only use the fewest measurements to get the best performance, but also maintain the best effects whether the signals are noisy or noiseless.

### 3.2. PAI Imaging of the Phantom

**Figure 4:**
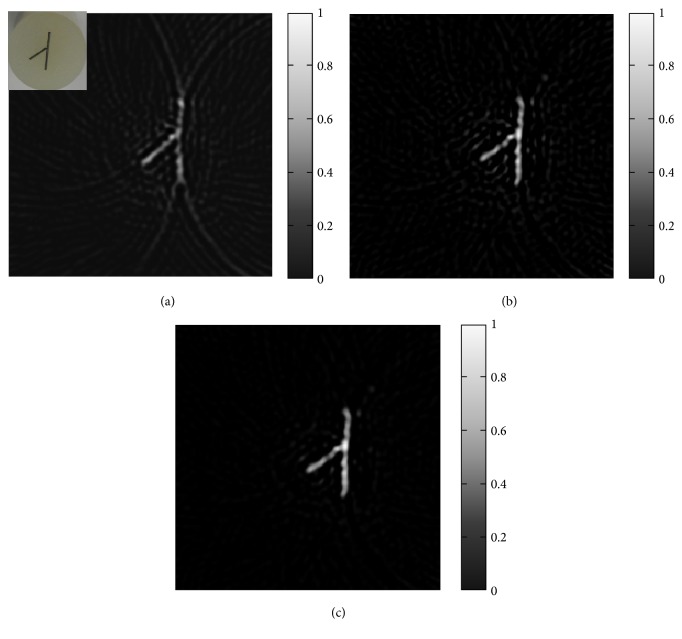
Reconstructed images based on few-view data. (a) 60 detectors over 180°, with FBP. The insert at the top-left corner is the photograph of the phantom. (b) 60 detectors over 180°, with IFBP. (c) 60 detectors over 180°, with RIWFBP.

In the phantom experiment, the RIWFBP method was tested and evaluated. For comparative purposes, reconstruction results of the FBP method and the IFBP method are also presented. Two graphite rods with a diameter *r* = 0.5 mm and lengths of 5 mm and 10 mm were buried in a cylindrical phantom at a depth of 4 mm. The phantom made of a mixture with 1% intralipid, 6% gelatin, and 93% water was used to simulate biological tissues. The inset in Figure 4(a) is the photograph of the phantom.

The sample was irradiated with pulses from a Q-switched Nd: YAG Laser. The pulse duration was 7.5 ns and the pulse repetition rate was 10 Hz. A focused hydrophone (Precision Acoustics Ltd.) with frequency response of 5 MHz was controlled by a high precision stepping motor to scan around the phantom in a circular manner for photoacoustic signal acquisition. The distance between the transducer and the rotation center was 45 mm. The induced photoacoustic waves were captured at 60 positions over 180°. At each position 50 signals were averaged. In real experiments, we did not know the true image, so the iterative stop criteria,

(12) $$\begin{array}{c} e_{i}=\frac{{\left\|x_{i}-x_{i-1}\right\|}_{2}}{{\left\|x_{i}\right\|}_{2}}<\varepsilon ,\;\left(i=1,2,\ldots ,n\right), \end{array}$$

were taken.

Figures 4(a)–4(c) show the reconstructed photoacoustic image utilizing the FBP method, the IFBP method, and the RIWFBP method, respectively. As expected, the image reconstructed with the FBP method contains artifacts and distortions. The artifacts in Figure 4(b) are much smaller than those in Figure 4(a). There is almost no artifact in Figure 4(c) which is in good agreement with the phantom. The results of the phantom experiment demonstrate that the RIWFBP method works well with few-view photoacoustic data.

## 4. Conclusions

In this work, the RIWFBP method has been applied to reconstruct photoacoustic images with few-view data. From the experiments we conclude that during the first five iterations, the RIWFBP method efficiently suppresses strips artifacts produced by IWFBP over 180°. The NMAE calculation also denoted that the RIWFBP method has an advantage in accuracy compared with other test methods. With regularization, the proposed method reaches the final solution faster than without. It is therefore easier to decide when to terminate the iterative loop. The application of the RIWFBP method will significantly reduce the number of ultrasound transducers and scanning time needed for high quality photoacoustic image reconstruction. Therefore, it can be a promising candidate for resolving the few-view PAI problem.
